# Retraction: Neonicotinoid’s resistance monitoring, diagnostic mechanisms and cytochrome P450 expression in green peach aphid [*Myzus persicae* (Sulzer) (Hemiptera: Aphididae)]

**DOI:** 10.1371/journal.pone.0272184

**Published:** 2022-08-03

**Authors:** The PLOS ONE Editors

The *PLOS ONE* Editors retract this article [1] because it was identified as one of a series of submissions for which we have concerns about authorship, competing interests, and peer review. We regret that the issues were not addressed prior to the article’s publication.

SS either did not respond directly or could not be reached. KM and MUS responded but expressed neither agreement nor disagreement with the editorial decision. ASA, KGR, and MH did not agree with the retraction.
